# Deep learning to overcome Zernike phase-contrast nanoCT artifacts for automated micro-nano porosity segmentation in bone

**DOI:** 10.1107/S1600577523009852

**Published:** 2024-01-01

**Authors:** Andreia Silveira, Imke Greving, Elena Longo, Mario Scheel, Timm Weitkamp, Claudia Fleck, Ron Shahar, Paul Zaslansky

**Affiliations:** a Department for Restorative, Preventive and Pediatric Dentistry, Charité-Universitaetsmedizin, Berlin, Germany; b Institute of Materials Physics, Helmholtz-Zentrum Hereon, Geesthacht, Germany; c Elettra – Sincrotrone Trieste SCpA, Basovizza, Trieste, Italy; d Synchrotron Soleil, Saint-Aubin, France; e Fachgebiet Werkstofftechnik / Chair of Materials Science and Engineering, Institute of Materials Science and Technology, Faculty III Process Sciences, Technische Universität Berlin, Berlin, Germany; f Koret School of Veterinary Medicine, The Robert H. Smith Faculty of Agriculture, Food and Environmental Sciences, Hebrew University of Jerusalem, Rehovot, Israel; Tohoku University, Japan

**Keywords:** Zernike phase contrast, X-ray nanotomography, deep learning, computer-aided image segmentation, lacuna-canalicular network, Sensor3D model, U-Net model

## Abstract

Bone material contains a hierarchical network of micro- and nano-cavities and channels, known as the lacuna-canalicular network (LCN), that is thought to play an important role in mechanobiology and turnover. The LCN comprises micrometer-sized lacunae, voids that house osteocytes, and submicrometer-sized canaliculi that connect bone cells. Characterization of this network in three dimensions is crucial for many bone studies. To quantify X-ray Zernike phase-contrast nanotomography data, deep learning is used to isolate and assess porosity in artifact-laden tomographies of zebrafish bones. A technical solution is proposed to overcome the halo and shade-off domains in order to reliably obtain the distribution and morphology of the LCN in the tomographic data. Convolutional neural network (CNN) models are utilized with increasing numbers of images, repeatedly validated by ‘error loss’ and ‘accuracy’ metrics. U-Net and Sensor3D CNN models were trained on data obtained from two different synchrotron Zernike phase-contrast transmission X-ray microscopes, the ANATOMIX beamline at SOLEIL (Paris, France) and the P05 beamline at PETRA III (Hamburg, Germany). The Sensor3D CNN model with a smaller batch size of 32 and a training data size of 70 images showed the best performance (accuracy 0.983 and error loss 0.032). The analysis procedures, validated by comparison with human-identified ground-truth images, correctly identified the voids within the bone matrix. This proposed approach may have further application to classify structures in volumetric images that contain non-linear artifacts that degrade image quality and hinder feature identification.

## Abstract 2

A deep-learning processing approach is proposed to assess in three dimensions the micro- and nano-porosity in bone imaged by Zernike nano-computed tomography.

## 1. Introduction

X-ray computed tomography (CT) bone research aims to non-destructively provide volumetric data with high resolution and contrast in order to reveal details of the internal structures of samples. This is particularly interesting for bony tissues, where the micro-geometry and internal porosity of blood vessels and cells are of paramount importance for disease investigation. Modern X-ray imaging systems have made significant progress since the first devices were developed half a century ago (Chen *et al.*, 2012 ▸; Ou *et al.*, 2021 ▸). They routinely provide valuable information about tissue density, morphology and sometimes dynamics, *e.g.* response to mechanical load (Kherlopian *et al.*, 2008 ▸; Weissleder & Nahrendorf, 2015 ▸). X-ray images (radiographs) can now achieve a spatial resolution better than ∼50 nm at high-flux synchrotron radiation facilities (Leake *et al.*, 2019 ▸; Martínez-Criado *et al.*, 2016 ▸; Quinn *et al.*, 2021 ▸). However, revealing details with sub-micrometer resolution usually requires some form of contrast enhancement since simple absorption differences and projection contrast are often poor (*e.g.* when differences in local composition are small) (Varga *et al.*, 2015 ▸; Zeller-Plumhoff *et al.*, 2021 ▸).

Phase-contrast enhancement (PCE) methods have been successfully implemented at many synchrotron facilities for some time now, taking advantage of the high X-ray photon flux as well as coherence (Wilkins *et al.*, 2014 ▸). Methods such as Zernike phase contrast shift the phase in parts of the incident light. Overlaps of non-shifted and shifted regions of the illuminated field yield constructive or destructive interference patterns that result in easy-to-detect intensity distributions (Momose, 2017 ▸; Pfeiffer *et al.*, 2006 ▸; Tao *et al.*, 2021 ▸). The result is that the contrast near structural inhomogeneities increases significantly (Holzner *et al.*, 2010 ▸; Zernike, 1942 ▸). This differs from standard X-ray absorption radiography, which is mainly sensitive to absorption of the transmitted X-rays that pass through the sample, where the contrast depends on the differences encoded in the imaginary part of the refractive indices. PCE methods merge the values for the real and imaginary parts of the refractive indices of sample components (Nave, 2018 ▸; Tao *et al.*, 2021 ▸). They are much more sensitive to internal defects and interfaces, but they yield data that have a non-trivial relationship to the density. Complementing other PCE nanotomography methods (*e.g.* Holo-CT) (Hesse *et al.*, 2015 ▸; Yu *et al.*, 2018 ▸), Zernike phase contrast overcomes the limitation of absorption contrast and achieves a very high resolution in rapid time by use of a transmission X-ray microscopy (TXM) configuration. Unfortunately, differences in the propagation paths across the field of view induce non-uniform gradients in the phase shift that produce extensive bright and dark distortions known as halo and shade-off artifacts (Kim & Lim, 2022 ▸). The size and intensity of these artifacts are both sample- and setup-dependent. For example, they are modulated by the characteristics (*e.g.* the width) of the phase ring that shifts parts of the phase of the diffracted wave (Yang *et al.*, 2014 ▸; Vartiainen *et al.*, 2014*a* ▸; Yin *et al.*, 2012 ▸). There have been proposals for software- and hardware-based approaches to eliminate these halo and shade-off artifacts in specific cases (Allan *et al.*, 2020 ▸; Kumar *et al.*, 2015 ▸; Vartiainen *et al.*, 2014*b* ▸; Yang *et al.*, 2014 ▸). However, no phase retrieval nor other generalizable solution has been found so far, in particular when attempting to resolve fine structural details such as the sub-micrometer pores found in tissue.

Sub-micrometer imaging is fundamental for assessing bone micro-morphology and understanding bone geometry and density changes over time (Müller, 2009 ▸; Akhter & Recker, 2021 ▸; Garnero *et al.*, 1996 ▸). Detailed three-dimensional (3D) information about the internal architecture is routinely collected by a range of methods sensitive to micro- and nano-porosity, including advanced optical techniques such as 3D-confocal imaging with fluorescent stains, focused ion beam electron microscopy (FIB-SEM) and micro- and nanoCT (van Tol *et al.*, 2020 ▸; Repp *et al.*, 2017 ▸; Hasegawa *et al.*, 2018 ▸; Palacio-Mancheno *et al.*, 2014 ▸; Goff *et al.*, 2021 ▸). Bone structure is characterized by interconnected porosities: in the vascular system, cavities are 20–40 µm in diameter and mainly include blood vessels. The lacuna-canalicular network (LCN) consists of a range of different voids (Cardoso *et al.*, 2013 ▸; Hesse *et al.*, 2015 ▸; McCreadie *et al.*, 2004 ▸; Currey & Shahar, 2013 ▸). The larger features of the LCN comprise ellipsoid lacuna, voids 5–20 µm in diameter in which osteocyte cells reside in the living bone. Cells are interconnected by 0.2–0.5 µm-diameter channels known as canaliculi. They establish communication paths between the bone cells (Robling *et al.*, 2006 ▸; Goodship, 1987 ▸) and, in fact, the entire LCN architecture is an open porosity structure, important for many functions of bone homeostasis. Osteocyte cells in the lacuna cavities of bone are thought to sense load and translate it into biochemical signals for bone remodeling (Bonewald, 2011 ▸). Studying the LCN architecture and porosity is therefore key to bone research, especially at the nanoscale (Silveira *et al.*, 2022 ▸), for which methods such as Zernike phase contrast show great potential.

**Figure 1:**
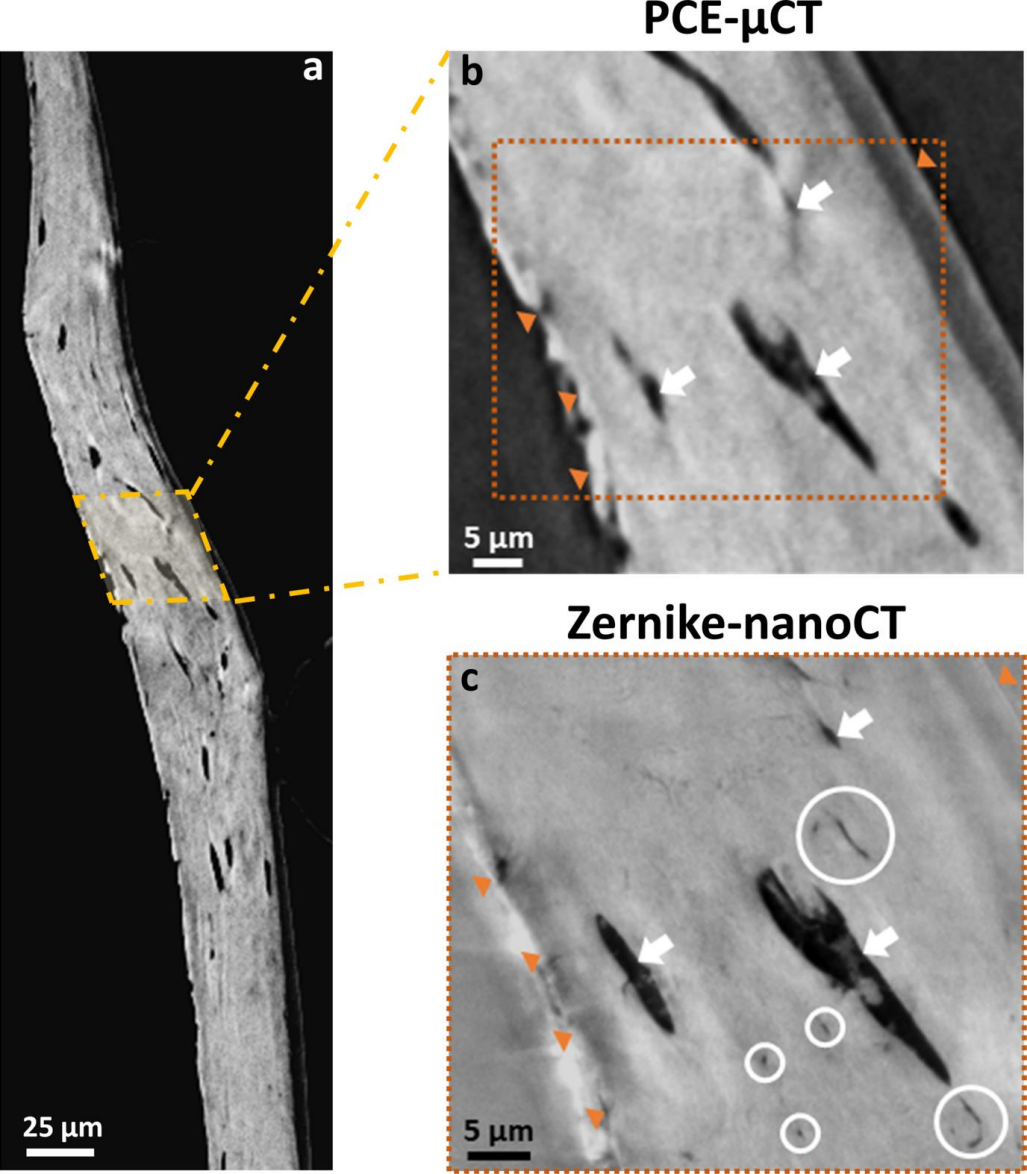
Examples of PCE reconstruction slices of zebrafish bones (*a*, *b*) propagation-based PCE-microCT versus Zernike-nanoCT (*c*). Both data types reveal the bone architecture (orange arrows) and micrometer-sized voids (white arrows). The Zernike-nanoCT reconstruction slice (*c*) unveils both the larger voids (white arrows) and smaller nano-porosity (white circles). Despite the increased contrast, Zernike-nanoCT becomes degraded by the appearance of halo and shade-off artifacts that are clearly identifiable at the edges of the sample (bright streaks and darker orange arrows). Some glue used for sample mounting is also visible on the outer bone surface on the right.

Synchrotron nanoCT has been popularized by its power to reveal the architecture of the inner porosity of bone at very high resolutions without the need for staining while providing volumes spanning tens of micrometers (Akhter & Recker, 2021 ▸; Stockhausen *et al.*, 2021 ▸; Larsson *et al.*, 2019 ▸; Takeuchi & Suzuki, 2020 ▸; Yuan *et al.*, 2012 ▸; Langer *et al.*, 2012 ▸). Coupling Zernike phase contrast (Zernike, 1942 ▸) to nanoCT provides rapid single scans of high-contrast images with spatial resolutions down to ∼100 nm (Flenner *et al.*, 2020 ▸; Longo *et al.*, 2020 ▸; Weon *et al.*, 2006 ▸). Figs. 1 ▸(*a*) and 1(*b*) illustrate the advantages and limitations of Zernike phase-contrast nanotomography (Zernike-nanoCT) by comparing it with PCE-microCT. Due to the enhanced contrast, Zernike-nanoCT [Fig. 1 ▸(*c*)] reveals features that are not visible in the PCE-microCT data [Figs. 1 ▸(*a*) and 1(*b*)], such as canaliculi (identified by circles). However, halo and shade-off artifacts also emerge, particularly near interfaces within the sample, introducing artificial gradients and ghost features (Vartiainen *et al.*, 2015 ▸; Yin *et al.*, 2012 ▸). Transforming such data into quantitative descriptors of the LCN architecture is challenging because classical segmentation/thresholding approaches to classify and separate bone material from air and cavities are not straightforward. Therefore, to maximize the benefit of Zernike-nanoCT imaging at fast acquisition rates, there is a need for automation approaches to analyze and successfully classify and process large quantities of such data.

The ideal automatic 3D image classification method should objectively identify voids and channels within the bone matrix. Objective, observer-independent protocols are needed to reach a reproducible quantitative and systematic assessment of the 3D morphology and topological attributes of the LCN. Emerging artificial intelligence approaches have the potential for performing automated segmentation and classification within variable contrast data. Deep learning (DL) models using convolutional neural networks (CNNs) provide new ways for the identification of visible features by means of trained image segmentation (LeCun *et al.*, 2015 ▸; Krizhevsky *et al.*, 2012 ▸). CNNs are important machine-learning models with internal architectures inspired by the biological process of neural networks and synapse formation (Alzubaidi *et al.*, 2021 ▸; LeCun *et al.*, 2015 ▸) and have many applications such as enhancing the visual quality of low-contrast images (Shelhamer *et al.*, 2017 ▸; Krizhevsky *et al.*, 2012 ▸; Liu & Zhang, 2018 ▸). Of the different architectures (Allan *et al.*, 2020 ▸; Sarvamangala & Kulkarni, 2022 ▸), the U-Net CNN is widely accepted as a standard for biomedical image classification due to the ability to recognize both large and small features under varied imaging conditions (Ronneberger *et al.*, 2015 ▸). Based on the U-Net architecture, the Sensor3D CNN model was designed to better segment organs in CT scans by making use of information in 3D (Novikov *et al.*, 2019 ▸). Importantly, the Sensor3D network is able to glean information from inter-slice correlations of the volume without requiring large amounts of memory. Both CNNs have a simple architecture and appear to reach high accuracy (Shen *et al.*, 2017 ▸; Brosch *et al.*, 2016 ▸; Zhang *et al.*, 2015 ▸; Horwath *et al.*, 2020 ▸; Wu *et al.*, 2013 ▸).

To use DL to identify features in tomographies, CNN models need first to be trained and then validated on reasonably large amounts of data (Alzubaidi *et al.*, 2021 ▸; LeCun *et al.*, 2015 ▸). Training requires human guidance to specify features of interest that must be correctly labeled. If multiple labeled images are made available for the CNN to classify different image regions, the model ‘learns’ features that match the corresponding labeled images (Bharadwaj, Prakash & Kanagachidambaresan, 2021 ▸; Krizhevsky *et al.*, 2012 ▸). It is convenient to train features of interest in 2D slices extracted from the 3D data, provided that, during ‘learning’, sufficient amounts of data are available to create internal maps that represent typical features of interest. The result of ‘learning’ is thus a series of weights (that convey ‘significance’ to recurring patterns) that can be tuned to outline defined features in a given dataset (Bharadwaj *et al.*, 2021 ▸; LeCun *et al.*, 2015 ▸; Shin *et al.*, 2016 ▸). Importantly, the data selected for training must be representative and contain all features to be identified in the tomography (Fang *et al.*, 2021 ▸; Gao & Zhong, 2020 ▸). It is common to name images in which a human operator identified features as ‘ground truth’, the term used for reference classification, that is considered to represent the ideal outcome (Zhou *et al.*, 2018 ▸). During training cycles, classification predictions are repeatedly compared with the ground truth, adding details that are needed to reach correct feature matching. The degree of match between classification predictions and the ground truth is evaluated by quantitative measures of error and accuracy (Kofler *et al.*, 2021 ▸; Setiawan, 2020 ▸). Error can be measured through ‘error loss’ functions of which the categorical cross-entropy is a widely used approach to measure the discrepancy between model predictions and ground truth (Bharadwaj *et al.*, 2021 ▸; Chen *et al.*, 2020 ▸). To evaluate the accuracy of the classification, a frequently used metric is the Dice coefficient, where a similarity metric is computed between model predictions and ground-truth images (Dice, 1945 ▸; Zou *et al.*, 2004 ▸). Model training ends after completion of a sufficient number of iterative cycles (epochs) so that low error loss is achieved. Dice coefficient and categorical cross-entropy can then be used to evaluate how good the training is and, in a second phase, to also validate the quality of the trained model by checking the classification on not-yet-analyzed images, for which additional ground-truth data are provided (Ali *et al.*, 2021 ▸; Ding & Möller, 2022 ▸). Training takes place by assessing small image regions, known as ‘patches’. Groups of patches from ‘batches’ of image regions are used as input to the neural network during training. The batch size refers to the number of patches processed in parallel during training. A larger batch size is able to achieve faster training with similar error loss as compared with smaller batch sizes; however, it tends to be less precise (Kandel & Castelli, 2020 ▸). Thus, a batch size of 64 results in faster training but with lower accuracy than a batch size of 32. Both batch and training data size (number of images to analyze) have an impact on the speed of model generation as well as on the accuracy of its prediction capacity (Kandel & Castelli, 2020 ▸; Kofler *et al.*, 2021 ▸). Typical CNN training uses 80% of the labeled ground-truth data for training, and 20% are later used for model validation. Since ground-truth data may be limited, computational approaches using data augmentation as well as transfer learning from pre-trained models are often used. Data augmentation techniques increase the variability and include random cropping, rotation, flipping and distortion (Mikołajczyk & Grochowski, 2018 ▸; Takahashi *et al.*, 2020 ▸). For transfer learning, a model that was trained on some previous, different dataset is reused and re-trained using a small dataset of new ground-truth images to perform a different classification task (Mustafa *et al.*, 2021 ▸; Yosinski *et al.*, 2014 ▸). Typically, a smaller data size is sufficient to reach robust learning, thus recycling the knowledge and resources (time, data) needed to train the CNN in the first place.

In this work, we describe and demonstrate a complete procedure for generating sufficient amounts of human-validated ground-truth images, for DL-assisted segmentation of internal bone porosity in noisy high-resolution Zernike-nanoCT data. Our proposed approach combines available libraries and image-processing tools to analyze LCN porosity in zebrafish bone, a popular animal model often used to study skeletal growth, bone disorders and drug development (Lin *et al.*, 2016 ▸). We explore the benefits of DL to find reasonable training parameters for the segmentation accuracy of U-Net and Sensor3D CNN models. Based on data obtained in two different Zernike-nanoCT synchrotron TXM setups and tested by transfer learning, we propose a detailed procedure that overcomes the inherent halo and shade-off artifacts in osteocyte-containing bone tomography data.

## 2. Methods

### 2.1. Materials

To train DL models to identify bone porosity, data from two synchrotron beamlines in France and Germany were used to generate Zernike-nanoCT reconstructions of zebrafish spine bones, known to contain an LCN. Cryopreserved vertebral columns of zebrafish were available from carcasses discarded as part of a previous study (Ofer *et al.*, 2019 ▸) with ethical approval from the Hebrew University of Jerusalem (permit MD-16-14844-3). Following removal of the soft tissue, the fifth caudal vertebra and spines (counted from the tail) were dehydrated in an ascending series of ethanol solutions (50%, 75%, 100%) followed by immersion in 100% acetone. Fig. 1 of the supporting information shows an example image of a typical caudal vertebra of a zebrafish skeleton.

### 2.2. Data generation: Zernike-nanoCT imaging and reconstruction

The hemal spine of each vertebra was mounted upright on a pin holder, fixed using light-curing transparent polymer. Multiple spine samples were imaged on the TXM branch of the ANATOMIX beamline at Synchrotron SOLEIL (French national synchrotron facility, Saint-Aubin, France). Other spine samples were imaged at the TXM endstation of the P05 imaging beamline of DESY, PETRA III (German national synchrotron radiation facility, Hamburg, Germany; operated by Helmholtz-Zentrum Hereon). The experimental setup of Zernike-nanoCT imaging beamlines comprises a beam-shaping condenser lens before the sample, a Fresnel zone plate behind the sample, a set of concentric Zernike phase rings to induce phase contrast, and a beam stop behind the beamshaper to protect the detector. For detailed descriptions of each of the beamlines used, see Flenner *et al.* (2020 ▸), Longo *et al.* (2020 ▸) and Scheel *et al.* (2022 ▸). A schematic of a typical experimental setup is given in Fig. 2 of the supporting information. For both beamlines, the true resolution of the images was 130–150 nm. The imaging conditions on the two setups were similar, with measurements on ANATOMIX performed at 10 keV and 1 s exposures, with a Hamamatsu Orca Flash 4 V2 2048×2048 CMOS-based camera, coupled via commercial microscope optics to a lutetium aluminium garnet single-crystal scintillator, whereas on P05 an energy of 11 keV and 86 ms exposure times were used with a Hamamatsu C12849-101U 2048×2048 pixel detector.

To unify data processing, the tomographic scans were normalized by flat-field correction (division by average empty beam images acquired at the beginning and the end of each scan) using a median filter with a radius of 2 pixels, applied to reduce noise. All tomographic reconstructions were performed using the back-projection method implemented in the *Nrecon* software (NRecon 1.7.1.0; Bruker micro-CT, Kontich, Belgium). Due to memory limitations of 3D DL data processing, the reconstructed volumes were cropped and binned with *Fiji* (Schindelin *et al.*, 2012 ▸), resulting in similar effective pixel sizes of 94 nm for ANATOMIX and 88 nm for P05. Each 3D dataset, therefore, comprised a total of ∼420 images (512×512 pixels), making it possible for the DL tools to reliably process the data within the available memory of 256 GB. Further analysis was performed employing the standard libraries of the *Dragonfly* 3D processing package (Dragonfly 2021.3; Object Research Systems, Montreal, Quebec, Canada) (Provencher *et al.*, 2019 ▸).

**Figure 2:**
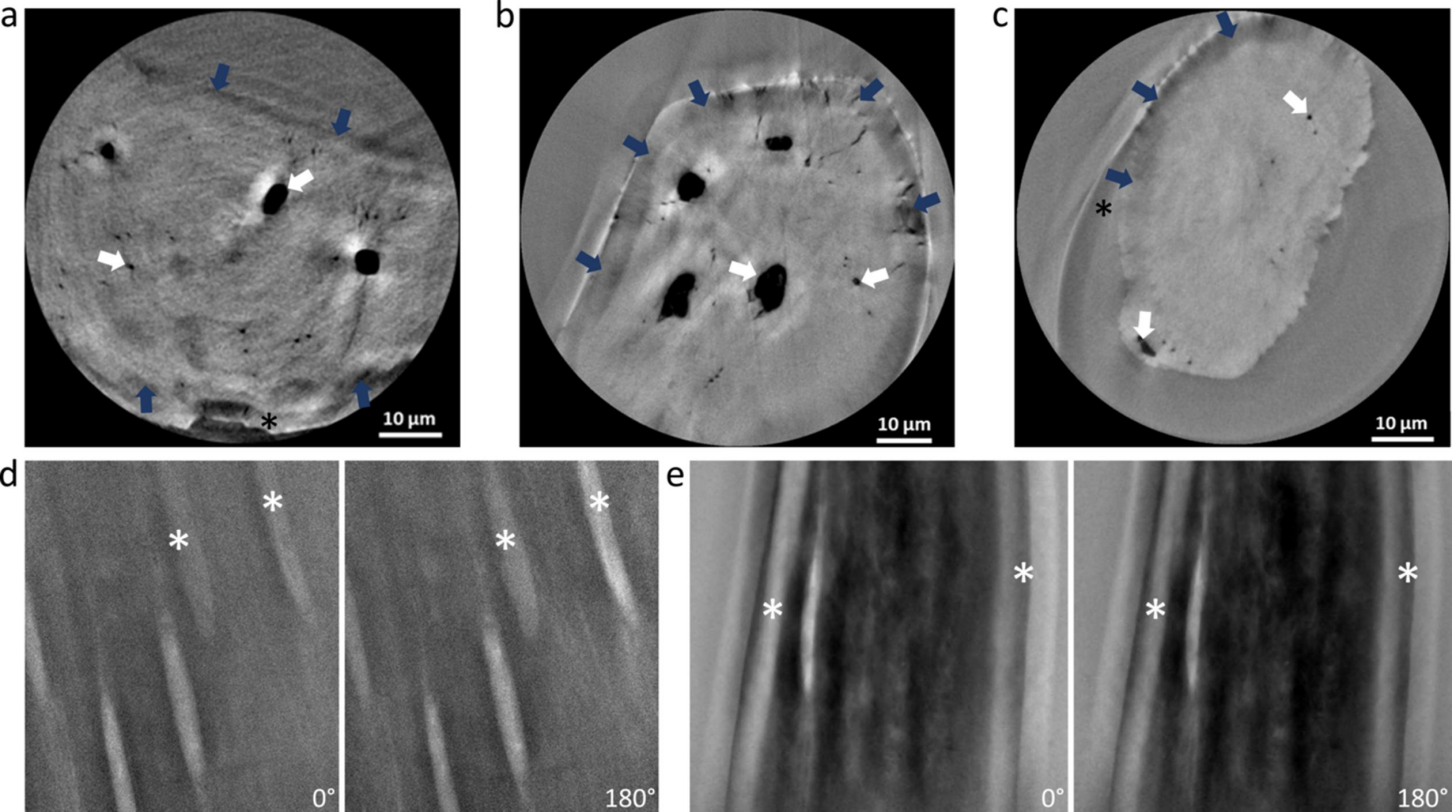
(*a*–*c*) Examples of reconstructed slices and (*d*–*e*) typical raw-data 0° and 180° radiogram images obtained from Zernike-nanoCT scans of zebrafish spines. Reconstructed slices in 3D data from P05 (*a*) and from ANATOMIX (*b*, *c*) beamlines contain prominent halo and shade-off artifacts (indicated by blue arrows). All reconstructions reveal the bone spine (large structure in the center), micro-voids and internal porosity (white arrows) that corresponds to the LCN typical of osteocytic bone. Black asterisks (*) indicate polymer-covered regions near the edges of samples (*a*) and (*c*). Halo and shade-off artifacts arise from a mixture of strong bright streaks or shadows due to interference effects at boundaries between different material densities mixed with variations in the contrast of such boundaries. The size and intensity of these artifacts emerge from a summation of different contrasts when the sample is radiographed from different perspectives. The 180° opposite (horizontally flipped) radiograms extracted from tomographic scans of two samples demonstrate how the contrast of identical features varies. White asterisks highlight regions that differ in contrast at 0° and 180° angles.

Examples of reconstructions of spine scans revealing bone, pores and edge-enhancing halo and shade-off artifacts are shown in Fig. 2 ▸. The variations of the contrast due to different interference effects observed when the sample rotates within the illumination field of the Zernike phase-contrast setup are demonstrated in example slices taken from scans on both P05 and ANATOMIX. The cause of such artifacts is the varying contrast of the same regions in different radiographs, as demonstrated in Figs. 2 ▸(*d*)–2(*e*), showing radiograms of the sample obtained at 0° and 180°. The same features are seen to appear with noticeably different contrasts. The white asterisks in Fig. 2 ▸(*d*) highlight regions with lower contrast in the 0° radiogram and higher contrast in the 180° radiogram, whereas Fig. 2 ▸(*e*) shows the opposite trend. Subsequently, reconstruction integrates all contrast variations so that the tomographic data exhibit, in addition to the true representation of the sample geometry, strong halo and shade-off artifacts that produce ghost features. In native bone samples, the shade-off artifacts are stronger in concave-shaped samples [Figs. 2 ▸(*b*) and 2(*c*)] than in broad sample geometries [Fig. 2 ▸(*a*)], and they are enhanced at interfaces with air. This differs from bone regions covered by polymer [black asterisks in Figs. 2 ▸(*a*) and 2(*c*)] due to reduced scattering and attenuated shade-off darker contrasts. The micro- and sub-micro-voids of the LCN are easily discernible to the human eye in the cross-sectional slices of the zebrafish bone material [Fig. 2 ▸(*a*)], though they do not have a unique gray shade.

### 2.3. Reference segmentation – ground-truth data

Usually, ground-truth data are carefully and painstakingly classified manually in 2D slices taken from the tomographic reconstructions, and they form the basis for CNN training. Therefore, 20 ground-truth images (5% of the entire dataset) were initially annotated by manually labeling multiple slices using the brush tools of *Dragonfly*. Different features were identified and assigned to one of several classes (bone, LCN, shade-off and background classes). The shade-off class corresponded to the darker region around the edges of the bone [blue arrows in Figs. 2 ▸(*b*) and 2(*c*)] for which an individual class was needed to identify the bone geometry properly. Though present throughout the data [Fig. 2 ▸(*a*) and 2(*b*)], halos do not prevent successful DL model training for pore identification. As halos do not affect the bone or pore size estimations, they were not classified as an individual class for model training. To create a larger cohort of 50 ground-truth slices (12% of the dataset volume), a Sensor3D model, trained on the annotated 20 ground-truth data images, was used to classify and generate ground-truth data. Each slice was inspected, and falsely identified regions were manually corrected by adding or removing badly identified domains using the *Dragonfly* brush tools. The process was repeated to create a larger training size of 70 slices (16% of the whole dataset). In total, three training data groups of increasing amounts of ground-truth data were used for testing the different DL parameters, as described below. The same data were also segmented conventionally using standard Otsu thresholding libraries (the background class was set to 0, the LCN class was defined in the range from 0.05 to 0.42, the shade-off class was defined from 0.43 to 0.53, and the bone class was defined for the range 0.54 to 0.89 based on visual inspection).

### 2.4. Deep-learning model training and segmentation

**Figure 3:**
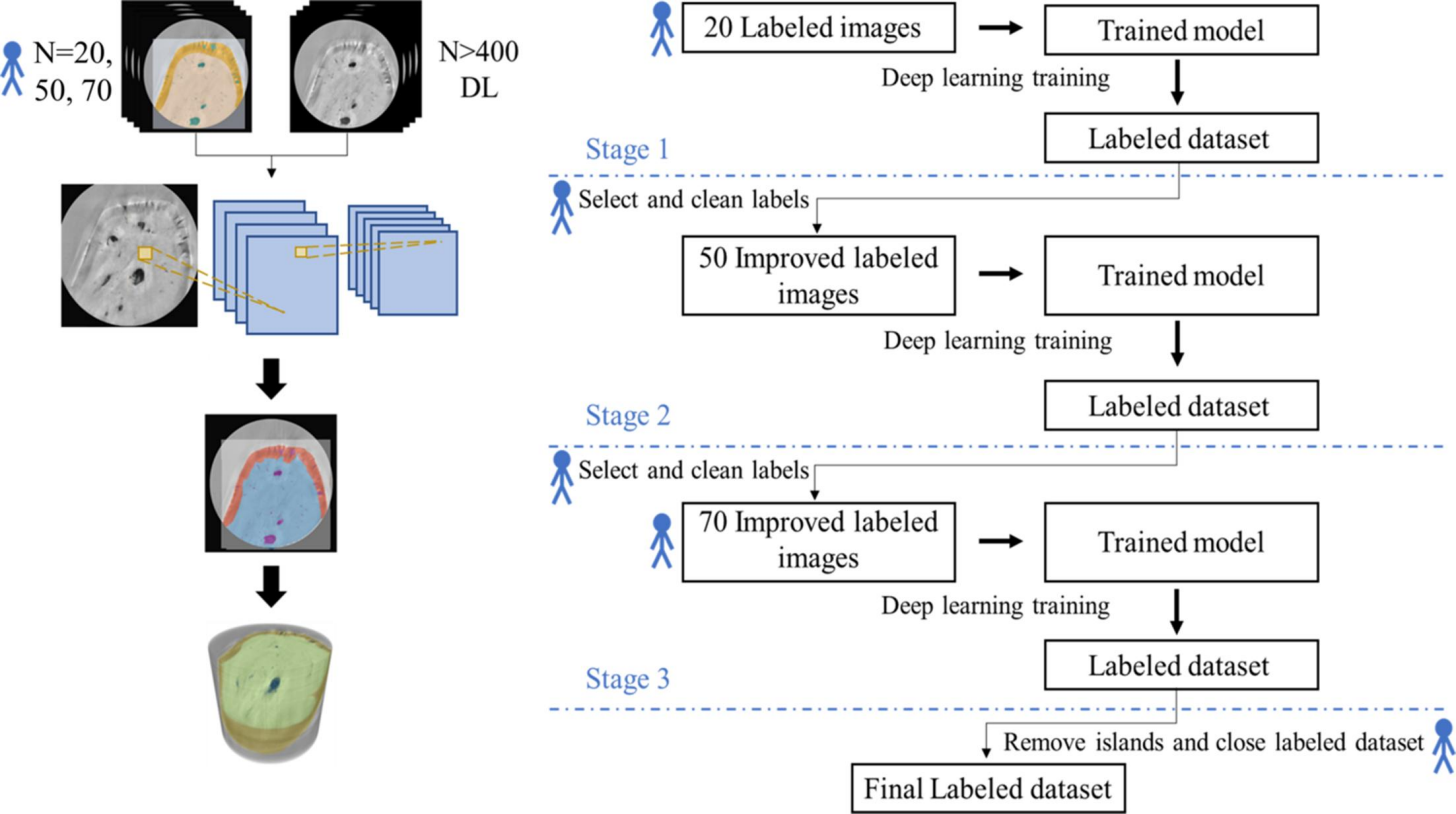
(Left) Typical process for ground-truth labeling then training of a CNN model for DL segmentation of Zernike-nanoCT imaged osteocytic bone. (Right) Flow chart of the generation of increasing numbers of ground-truth data. Stage 1 includes manual segmentation of 20 labeled images (ground-truth data) from the input 3D dataset, used to train and validate each of the Sensor3D and U-Net models. In stage 2, the trained Sensor3D model was used to segment 50 images from 3D data. These roughly segmented images were corrected for mislabeled pixels and used as new data to train and validate new Sensor3D and U-Net models. In stage 3, the trained Sensor3D model was used to segment 70 images from 3D data. These labeled images were corrected and selected as ground-truth images, used to train and validate new models. The final labeled results including bone, shade-off and LCN classes were filtered using the ‘remove islands and closing’ morphometrics operations.

Two CNN model architectures and several training parameters were tested to segment reconstructed data, using established tools. The DL models were trained to identify small and large internal pores as well as the outer geometry of the bone, in order to separate them from overlapping shade-off and halo artifacts. To achieve this, multiple U-Net and Sensor3D CNN models were trained on the three ground-truth (training data) sets using batches of 32 or 64 (batch size). Figs. 3 and 4 of the supporting information show schematic diagrams of the U-Net and Sensor3D architecture, respectively.

The annotated ground-truth data were used to both train and later evaluate the accuracy of classification in each of the models. Each model was trained on 80% of the total number of ground-truth images, with the remaining 20% used for model validation, checking the correspondence between prediction images with ground-truth images. All experiments were conducted using standard Adadelta optimization, a stochastic gradient descent method that adapts learning rates, and were computed on an NVIDIA GeForce RTX 2080 Ti graphic card with 4352 CUDA cores. Each model was trained for up to 100 epochs (number of data training computation cycles) until the cross-entropy loss function did not decrease by more than 0.01% for ten consecutive epochs. Each of the trained Sensor3D and U-Net models was used to segment the whole 3D dataset into the four defined classes (schematically outlined in Fig. 3 ▸).

To reveal the trend between training data size and the evaluation metrics, two intermediate, additional data sizes of 10 and 30 ground-truth images were used to train U-Net and Sensor3D models. In total, ten U-Net and ten Sensor3D models were trained on the five ground-truth sets using two batch sizes. The model trained with ANATOMIX data scoring the highest accuracy (Dice coefficient metric) and lowest error loss (categorical cross-entropy error loss function) was also used to segment the P05 dataset. This CNN was then also used as a pre-trained model, one that already has weights and ‘previous knowledge’ from the ANATOMIX data, for transfer learning. Consequently, it was trained to analyze the P05 data on 20 ground-truth images that refined the pre-trained model.

### 2.5. Image analysis

The segmented data were further processed using the ‘remove islands and closing’ morphometric operations to remove the ∼2% remaining mislabeled pixels. The shade-off and bone classes were merged in 3D since the shade-off class overlapped with the bone class. The background class that defines the outer bone geometry was used to crop the volume prior to further bone porosity analysis. Volume quantification of both the bone and LCN classes was performed using the *Dragonfly* ‘multi-ROI analysis’ module. Bone porosity was determined from the ratio of the LCN and bone volumes. LCN thickness was computed in 3D using the ‘Volume thickness map’ function. The LCN was then further analyzed by separating lacuna along connected canaliculi. Each cell and its respective connected units/voxels were converted into isolated regions of interest (ROI) using the ‘new multi-roi (26-connected)’ function in which units smaller than 100 voxels were discarded, assuming they arise from noise or are far smaller than typical lacunae and canaliculi. The volume of each isolated cell was then computed in 3D using the ‘multi-ROI analysis’ function.

### 2.6. Statistical analysis

Statistical analyses were performed using *RStudio* (RStudio Version 1.3, RStudio, PBC, Boston, MA, USA) to evaluate correlations between the trained CNN models, batch and training data sizes. The Wilcoxon signed-rank test was applied to compare the accuracy and error loss metrics between the Sensor3D and U-Net models. *P*-values < 0.05 were considered statistically significant. Correlations between the batch size and training data size parameters and the accuracy of the models were quantified using Spearman’s rank correlation method.

## 3. Results

### 3.1. Non-linear intensity contrasts of similar structures

**Figure 4:**
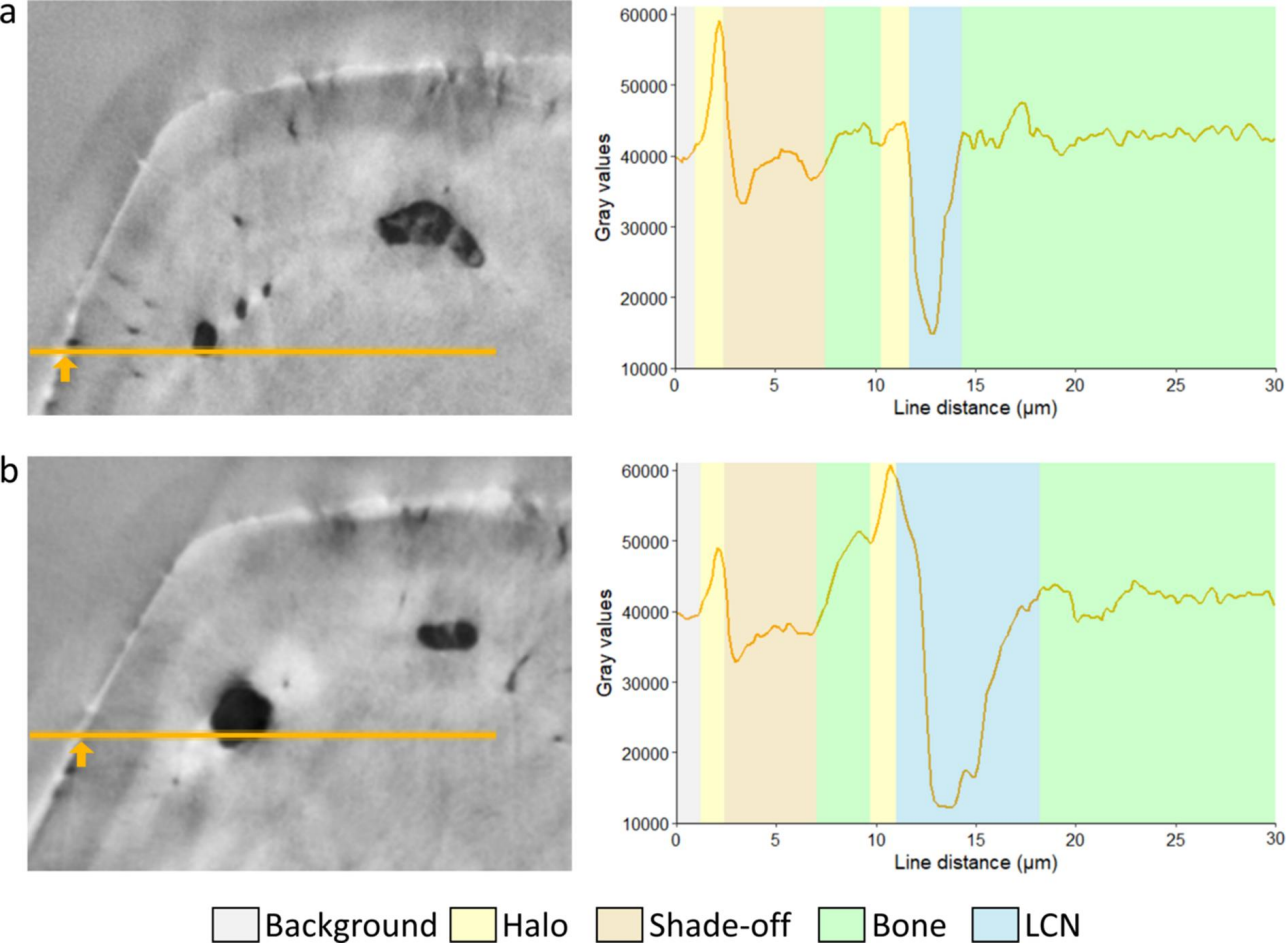
(*a*, *b*) Examples of slices within Zernike-nanoCT data scanned on ANATOMIX. Line profiles (shown in orange in panels *a* and *b*) across the same lacuna at different heights reveal the gray-level profile along the sample. Orange arrows identify the outer edges of the bone sample. Halo regions in (*a*) and (*b*) have higher values compared with the surrounding, though different absolute values (as seen by the peaks shaded in yellow) and are located around the edges of the sample and near the lacuna void (peak shaded in blue). The values of the outer-margin halo peaks vary, and are lower in (*a*) than in (*b*) whereas the internal halos in the same lines show an opposite trend. Shade-off regions (indicated in light brown) are seen around the edges of bone and vary significantly between slices (*a*) and (*b*). The bone matrix far from edges or pores (shaded in green) has an intermediate gray value.

The 3D reconstructions of Zernike-nanoCT of zebrafish bone spines exquisitely reveal bone porosity including lacunae and canaliculi, though with variable local contrasts (Figs. 1 ▸, 2 ▸). Numerous lacunae are visible within the scanned regions that exceed 48 µm^3^. Examples of different cross-sectional slices within a single reconstructed tomographic dataset imaged on ANATOMIX (Fig. 4 ▸) illustrate the typical bone features including osteocytic lacunae and canaliculi. The line profiles in Fig. 4 ▸ exemplify the non-linear gray values corresponding to distinct features of interest. The contrast enhancement created by the Zernike configuration comes at the price of creating significantly visible artifacts. Patterns of halos are seen on the sample edges (indicated on the line profiles by a yellow region) and they also overlap the bone material surrounding voids. Line profiles, indicated in orange and plotted on the right (of panels *a* and *b*), reveal that no single range of gray values can be used to uniquely identify a particular material (bone, void/air), as seen by comparison within and between virtual slices of the 3D data. The line profiles reveal a sharp peak in intensity (halos, yellow shaded regions in the plot) on the outer bone rim (orange arrows) and near voids. Shade-off effects produce a darker region around the sample edges (light brown shaded region in the plot). The strength of these artifacts varies in different virtual slices along the sample, *e.g.* halos seen around the lacunae are more intense in Fig. 4 ▸(*b*) than in Fig. 4 ▸(*a*). The contrast between shade-off regions near sample edges and the bone also fluctuates between the slices. Typical for the ellipsoid lacunae, the lateral extent of the osteocyte changes along its axis. However, the gray values also differ, at the margin between air (light-blue shaded region in the line plot) and bone (green shaded region in the line plots). Notably, even within the bone, a range of gray values is seen and often they are strongly affected by the proximity to the sample edges.

### 3.2. U-Net versus Sensor3D models

**Figure 5:**
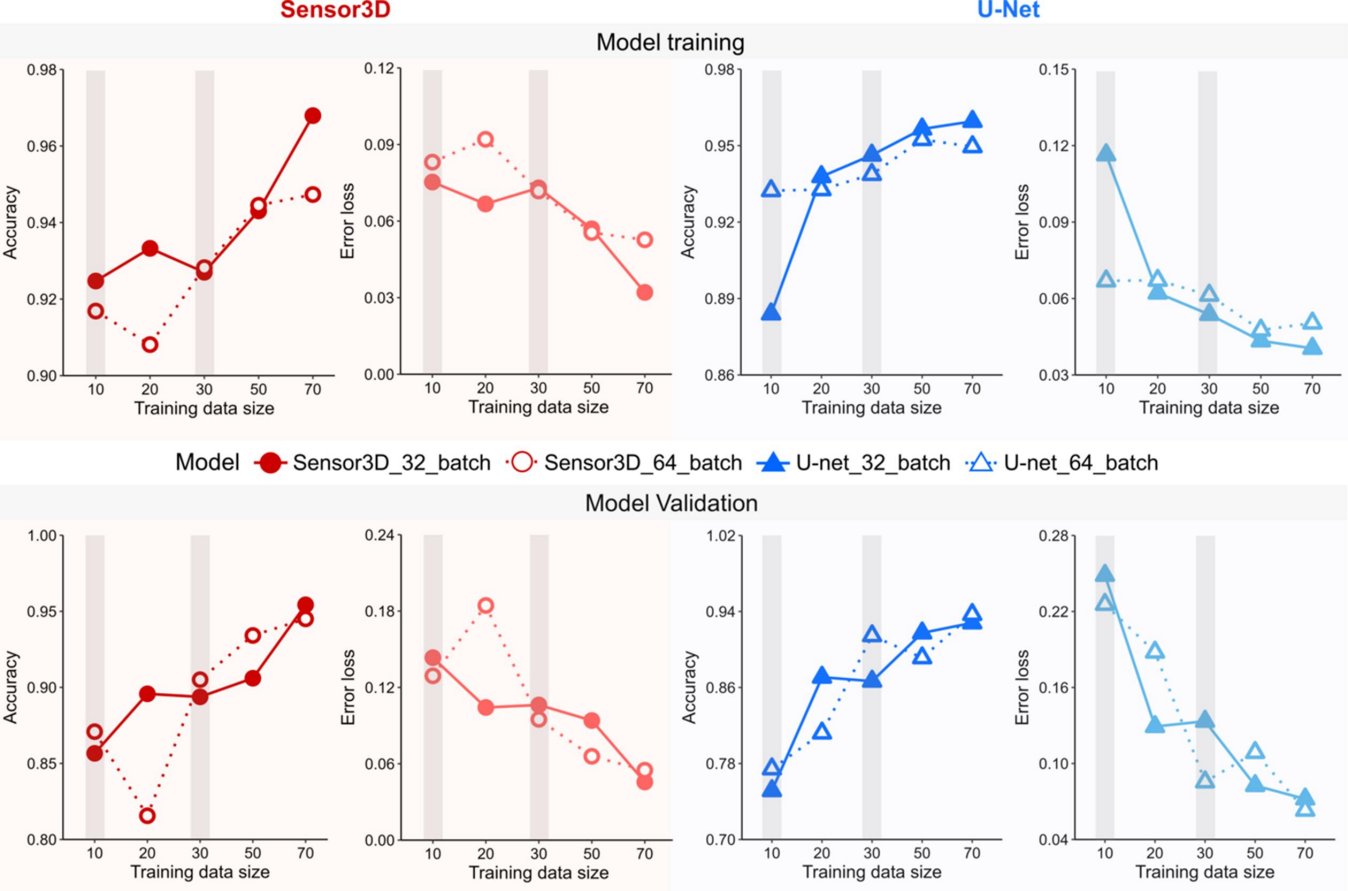
Analysis of the accuracy and error loss metrics for training and evaluation of ten Sensor3D and ten U-Net models. For most of the models, accuracy improves and the error decreases when increasing the number of training images. The main effect of the batch size is observed for models trained with a smaller training data size of 10 and 20, though the difference is not statistically significant. Whereas the Sensor3D model with a batch size of 32 and training size of 10, 20 and 30 required 71, 67 and 52 epochs, respectively, unexpectedly for a training size of 50, only 30 epochs were needed due to a lack of any increase in learning rate, whereas with 70 training images a total of 97 epochs were needed. For the Sensor3D model with a batch size of 64 and training sizes of 10, 20, 30, 50 and 70, the DL process required 65, 30, 45, 56 and 90 epochs, respectively, thus increasing with increasing training data sizes. The U-Net model with a batch size of 32 and a training sizes of 10, 20, 30, 50 and 70 images required 30, 43, 45, 56 and 51 epochs, respectively. The U-Net model with a batch size of 64 and training sizes of 10, 20, 30, 50 and 70 images were completed within 65, 25, 119, 55 and 65 epochs, respectively, again demonstrating that the number of epochs required is not linearly related to the training data size. The two intermediate additional data sizes of 10 and 30 ground-truth images are marked by gray shade regions.

The training efficiency of U-Net and Sensor3D models can be assessed by comparing the effects of changing the training data size and batch sizes. A reasonable balance was sought between the extent of data needed for training and validation versus training parameters affecting computation time and model accuracy (*e.g.* batch size). Fig. 5 ▸ compares the accuracy and error loss metrics for the multiple U-Net and Sensor3D CNN models, trained with the same data (correlations between the different parameters is given in Fig. 5 of the supporting information). For both CNN model types, the accuracy of models with 32 batch sizes increases and the error loss decreases with increased training data sizes (0.95 correlation, *p* < 0.05, see Fig. 5 and Tables 1 and 2 of the supporting information). The same is seen for the U-Net model with a batch size of 32, whereas, unexpectedly, the U-Net model with a batch size of 64 decreases in accuracy and increases in error loss during training with larger data sizes of 70 images. Yet when validating the model (see lower panels of Fig. 5 ▸), analysis shows that this model actually reached higher accuracy and lower error loss than the U-Net model trained with a data size of 50 images. We found no correlation between the accuracy metric and the batch size (*p* > 0.05, Fig. 5 of the supporting information). Models with a batch size of 64 took 2×–3× less time to train (larger batch size requires fewer iterations) than the models with a batch size of 32. However, in some cases, models trained with a batch size of 32 required fewer epochs (number of times that a model was trained) and therefore need shorter training durations than models with a batch size of 64 (see Fig. 6 of the supporting information for training durations between the different stages). Most U-Net and Sensor3D models have high accuracy (∼0.9) and low error loss (∼0.1) except for U-Net and Sensor3D models with a batch size of 64 and training data size of 20. No significant differences were found (*p* > 0.05) when using either accuracy or error loss as metrics. Of all the model training performed, the Sensor3D model with a batch size of 32 and a training data size of 70 images had the highest accuracy and the lowest error loss when evaluating the training data. Numerical training and validation results for the U-Net and Sensor3D models are provided in Tables 1 and 2 of the supporting information and a visual comparison between the different stages is provided in Fig. 7 of the supporting information.

**Figure 6:**
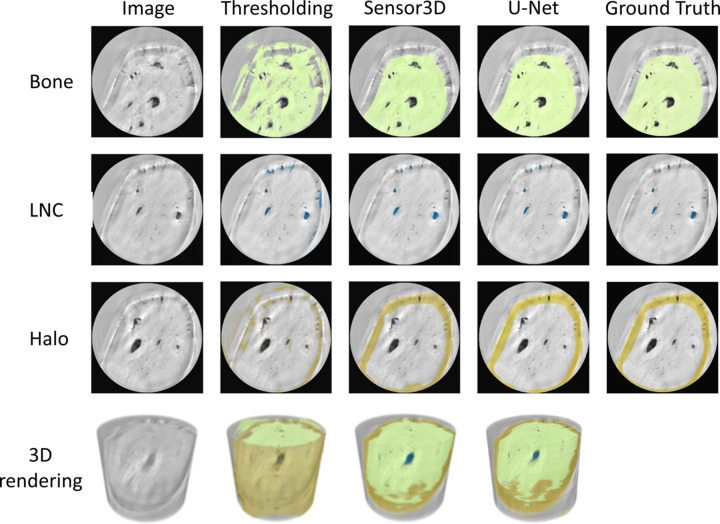
Example images and classification for shade-off (brown), bone (green) and the LCN (blue) classes performed on an ANATOMIX dataset comparing the result obtained with Otsu thresholding, the Sensor3D model with 32 batch size and 70 training size, the U-Net model with 64 batch size and 70 training size versus manual segmentation (ground truth). The standard Otsu thresholding has the worst outcome, mislabeling the bone class with background values because gray value segmentation yields ambiguous results. Both Sensor3D and U-Net models can correctly segment the voids, bone and shade-off regions which Otsu thresholding and other simple segmentation methods cannot.

Comparisons of segmentation outcomes from bone and voids by manual labeling, by Otsu thresholding, as compared with trained CNN models provide insights into the robustness of the DL training, especially in slices within entire tomographic reconstructions. Fig. 6 ▸ shows bone, LCN and shade-off classes in exemplary slices, along with 3D renderings of classified data segmented by Otsu segmentation as well as U-Net and Sensor3D models. We observe that some regions containing air outside of the bone were incorrectly classified as being bone by the U-Net model. Fig. 8 of the supporting information shows 3D renderings of classified data presented in Fig. 6 ▸ magnified. The standard Otsu threshold method applied to the whole sample yields highly unreliable results and exemplifies the difficulty in segmenting porosity data based solely on global gray values.

**Figure 7:**
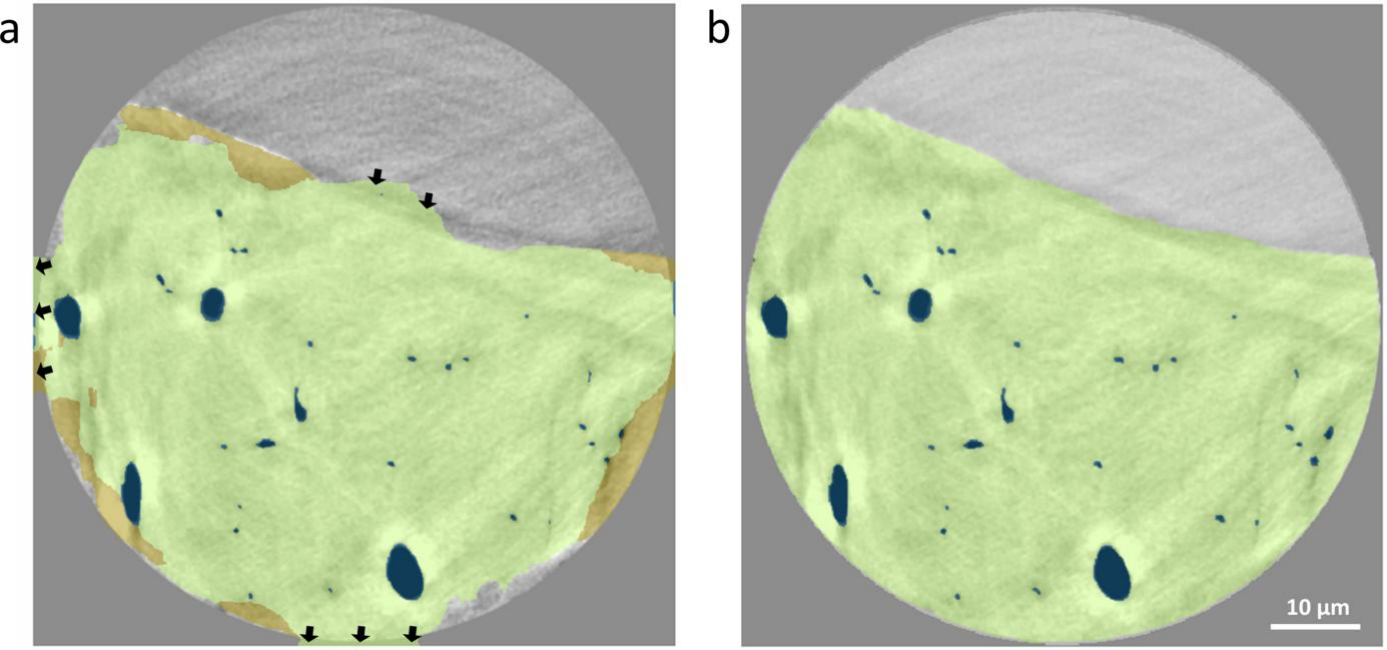
An identical cross-sectional slice of a zebrafish bone scanned in the Zernike-nanoCT setup of P05, labeled using the (*a*) Sensor3D model trained for the ANATOMIX dataset with 70 images and 32 batch size and applied to P05 data versus (*b*) the same model re-trained on 20 ground-truth images of the P05 dataset. Note that these data have different size, noise level and contrast range as compared with the ANATOMIX data. The as-trained model produced a compromised, though not totally incorrect, classification, mainly mislabeling edges (indicated by the black arrows and green regions) of the bone class that are incorrectly classified as background values. Whereas Sensor3D training on 20 images from scratch required 67 epochs, the use of a pre-trained ANATOMIX model, retrained with 20 ground-truth images of a P05 data, improved classification of both bone and LCN yet required only 37 epochs (1 h).

Transfer learning across the different datasets was explored using the best-trained ANATOMIX model (highest accuracy and lowest error). The Sensor3D model trained with 70 images and a batch size of 32 was used to train and segment a dataset of similar fishbone scanned on P05. The performance of the same model with no further training was compared with the classification performed with additional training using only 20 images as training data. By using the pre-trained CNN, rapid convergence was achieved, reaching sufficient training within ∼40 epochs. The segmentation results of Sensor3D model segmentation in classifying voids along the spine bone are shown in Fig. 7 ▸. Classification of the cross-sectional slices of zebrafish bone scanned on P05 shows that the pre-trained model that was re-trained with P05 images can better identify the boundaries of both LCN and bone [Fig. 7 ▸(*b*)] as compared with the same model which was only trained with ANATOMIX images [Fig. 7 ▸(*a*)]. Note that, for re-training, only three classes were used (bone, LCN and background) instead of four (bone, LCN, halo and background) due to contrast differences caused by shade-off artifacts between ANATOMIX and P05 datasets. This shade artifact is less pronounced in the P05 data than in the ANATOMIX data; therefore the Sensor3D model trained for the ANATOMIX data incorrectly detects areas of shade-off around the edges of bone that do not exist in the P05 data.

## 4. Discussion

Our results show how incremental DL training applied to reconstructions of Zernike phase-contrast enhanced tomography can reliably and predictably classify the micro-nano scale pores revealed in bone. The Zernike-nanoCT scans were reconstructed from enhanced contrast radiographs collected on different TXM machines. The features revealed are the main components of the LCN, with the resolution limit of the setups estimated to be in the 130–150 nm range. Consequently, our data reveal the major component of the porosity in osteocytic bone, though not smaller-scale nano pores (Tang *et al.*, 2022 ▸). A major advantage of the Zernike-nanoCT method is that bone segments can be imaged rapidly, within a relatively large field of view (∼48 µm^3^) and this approach makes it possible to repeat measurements on overlapping regions of interest so that even larger samples can be mapped rapidly in 3D. The samples are not destroyed during the imaging process, which is an important advantage over nano-resolution slice-and-view (FIB-SEM) analysis methods that are often used in bone research (Weiner *et al.*, 2021 ▸). In the experiments reported here, data were collected from two Zernike phase-contrast beamlines with similar setups. In both, a recurring disadvantage of these data is the appearance of halos and shade-off artifacts, as shown in Figs. 1 ▸, 2 ▸ and 4 ▸. Halo and shade-off artifacts are enhanced by cross-talk between the sample microstructures and the phase ring as well as the outer sample geometry (Vartiainen *et al.*, 2015 ▸). For example, concave sample regions exhibit strong shade-off artifacts as compared with broader samples [Figs. 2 ▸(*b*) and 2(*c*)]. Darker shade-off regions are very prominent at bone interfaces with air such as near lacunae, as opposed to bone regions covered by sample-gripping polymer, where interference effects are attenuated. We note the important difference in addressing noise versus image-artifacts: DL methods have previously been applied to denoise TXM data. However, it was shown that such DL denoising enhances halo artifacts in Zernike phase-contrast data, reducing the applicability for imaging the LCN in zebrfish bones (Flenner *et al.*, 2022 ▸). Such shade-off artifacts make segmentation of the bone data challenging since traditional threshold methods, which separate gray values and classify structures based on intensity, fail to reliably separate the voids and outer edges of the bone, as shown in Fig. 6 ▸. Comparisons between outcomes of DL segmentation show that the CNN models are excellently reliable alternatives to traditional segmentation methods conventionally used for the analysis of absorption contrast data. The results show that in all cases the Sensor3D models are slightly more accurate than U-Net for outlining the boundaries of the bone and LCN classes within the limits of the training data and batch sizes tested. This comes at a computational price in that it takes longer to train these CNN models as compared with U-Net models.

The ground-truth data size used for CNN model training can affect the classification of 3D tomographic reconstructions, especially with classes having variable contrast. For both the U-Net and Sensor3D models, incremental exposure to increasing amounts of training data improved the accuracy, resulting in an increased capacity to reliably classify structural features within the Zernike-nanoCT data. Since there is no way to know *a priori* which CNN model is better suited to robustly identify pores, bone and halo/shade-off artifacts, we compared the outcomes of model training and validation with different training data and batch size parameters. However, there are infinite possible parameter combinations and no universally prescribed guidelines regarding the number of ground images for CNN model training. Further, we want to avoid over-training. Therefore, the present study targeted finding a reasonable amount of training data that reliably yield a reproducible workflow. With this approach, the LCN pores in bone imaged by Zernike phase contrast were retrieved, a challenge of great relevance in many ongoing studies (Busse *et al.*, 2010 ▸; Cardoso *et al.*, 2013 ▸; Sharma *et al.*, 2012 ▸; Tommasini *et al.*, 2012 ▸). The U-Net and Sensor3D models mostly have high accuracy (∼0.9) and low error loss (∼0.1) except for models with a batch size of 64 and training data size of 10 and 20. Our results suggest that the outcome of classification by both CNNs excellently matches the labeling in the ground-truth images if the models have a training data size of 50 or more images. The Sensor3D model with a batch size of 32 and the larger training data size of 70 images had the highest accuracy and the lowest error loss (Fig. 5 ▸) for both training and validation (when ground-truth images are iteratively compared with model predictions of classification). Visual inspection of the outcomes of segmentation with both model types (Fig. 6 ▸ and also Fig. 8 of the supporting information) shows that the U-Net model yields larger numbers of mislabeled pixels, as compared with the Sensor3D model.

Increasing the batch size allows to speed up training. Most of the models with a batch size of 64 completed training in about half the time as compared with models with a batch size of 32. We found no correlation between the batch size and any of the accuracy and error loss metrics (Fig. 5 of the supporting information). This suggests that the determination of the optimal batch size parameter should be based on technical consideration of speed and memory and is not likely to affect the quality of the classification outcome. For transfer learning assessment, the model with a batch size of 32 was chosen due to slightly higher accuracy, though also training models with a batch size of 64 can be used when memory and time are limited. Our incremental approach creates a way to provide large amounts of ground-truth data so that any model can better ‘learn’ to identify porosity and other bone features (Fang *et al.*, 2021 ▸), reducing human-based and time-consuming classification efforts. Training data need to be representative and diverse enough so that the model can correctly identify recurring patterns and variable relationships between features with different contrasts in structures of the same type. This was not possible with training data sizes of 20 and 50. Incremental training stages make use of labeled data that were segmented with the help of intermediate rudimentary models to create a larger number of training images that is sufficient to train the CNN models reliably. In the initial stage, it is recommended to begin with a modest number of ground-truth images (between 10 and 20), depending on the data complexity. Subsequently, additional ground-truth images should be progressively incorporated based on the accuracy and error loss metrics. More stages will be needed if the accuracy and error loss values do not sufficiently improve. For Zernike-nanoCT reconstructions, this approach reduces the labor-intensive manual labeling efforts and shortens the time needed to achieve robust CNN models.

The U-Net and Sensor3D models were compared by accuracy and error loss metrics applied to both the training and validation steps. This approach provides an observer-independent evaluation of how accurately the trained models identify features in slices in the 3D data. An ideal model would generate predictions identical to the ground-truth labels in all slices. Practically, however, the model will never classify features as precisely as those marked in the ground-truth data since slices will have some noise and statistical error as well as some possible labeling bias. Additional available morphometric operations such as ‘remove islands and closing’ should be used to correct mislabelled porosity pixels.

It was also possible to use the Sensor3D model trained for the ANATOMIX dataset as a pre-trained model for successful segmentation of the P05 dataset, following additional training. Transfer learning is of increasing interest in building libraries of validated models that can be reused in other data (Rawat & Wang, 2017 ▸). Models that are trained for a specific task on one kind of data can suffer significant error loss when used on new data, as in the case shown in Fig. 7 ▸(*a*). However, in our data, excellent classification was achieved by retraining the model, used as a pre-trained model, a strategy that significantly shortens the learning process. Transfer learning is increasingly applied for medical imaging, especially when the trained models become too specialized (Matsoukas *et al.*, 2022 ▸; Mustafa *et al.*, 2021 ▸; Yosinski *et al.*, 2014 ▸). Accurate classification of new tomographic data does not require training from scratch, which significantly reduces training time and effort. Note that, due to different statistical noise and contrast, the Sensor3D model trained for the ANATOMIX dataset was only partially useful to segment the P05 dataset when used without further training. Contrast differences induced by shade-off artifacts are stronger in the ANATOMIX dataset than in the P05 dataset, making it difficult to segment the bone regions, since the CNN was not previously trained on such subtle but important detail. Both P05 and ANATOMIX data reveal important contributions of sample geometry (concavity, convexity) as well as the presence of mounting polymer, on the outer bone surface, as strong modulators for the prominence of shade-off artifacts, which was much less visible in the sample imaged on P05. Results suggest that transfer learning between different beamlines can be used, though the models require additional training, to reduce the efforts in reaching reliable Zernike-nanoCT segmentation.

In this study, Zernike phase-contrast data were reconstructed with a well established FBP algorithm, a widely adopted method in X-ray microtomography instruments due to its proven ability to deliver high-quality reconstructions across a wide range of sample types. Advanced reconstruction algorithms tailored to a given sample could have been a promising alternative. However, these alternatives often demand significantly greater computational resources, making them less practical for general use across various sample types. The proposed approach has the merit of being based on reconstruction tools readily available at the beamlines, making it versatile and applicable to a diverse array of sample types.

### 4.1. Examples of 3D assessment of DL segmented osteocytic bone data and LCN

**Figure 8:**
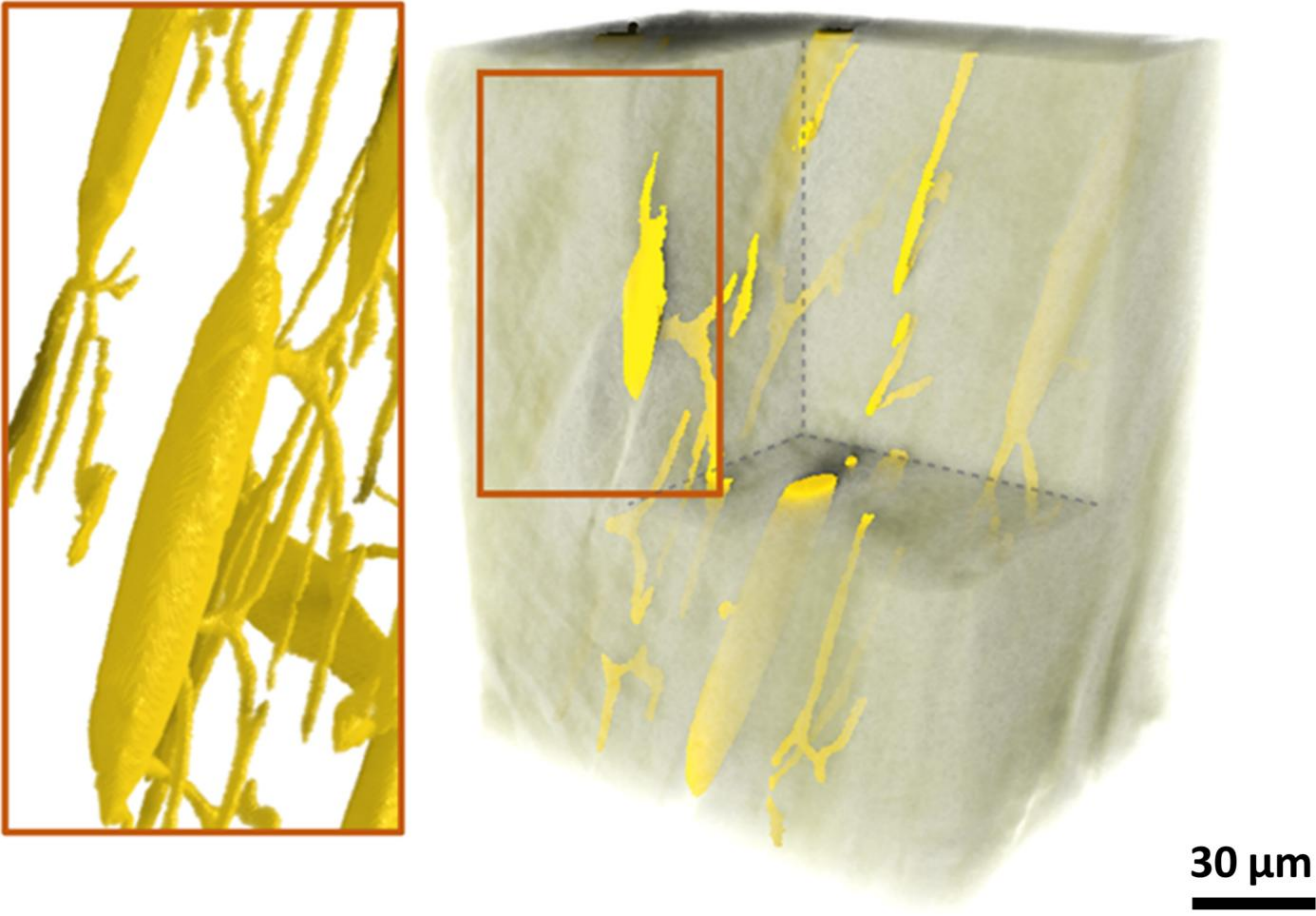
3D-rendering overview of the LCN porosity within zebrafish bone in an ANATOMIX dataset, segmented with the Sensor3D model. Analysis revealed an average porosity of 2.1%. Several canaliculi connecting to lacunae are seen in detail. Due to resolution limits, only canaliculi larger than 130 nm are visible.

Osteocytes and canaliculi (>150 nm diameter) of the LCN within the imaged bones are revealed, unstained, by Zernike-nanoCT imaging. The segmented LCN porosity, graphically rendered in Fig. 8 ▸, consists of a complex interconnected porous system that is typical for many osteocyte-containing bones. This segmentation was obtained with the Sensor3D model (batch size of 32 and training data size of 70 images) because of its higher accuracy and lower error loss compared with the other trained models. The lacunae appear as nodes connected through the canaliculi and are thought to enable communication between neighboring bone cells. The LCN class analyzed in 3D for bone scanned on ANATOMIX (data shown are for the Sensor3D DL analysis) has an average porosity of 2.1%, which is within the range of porosities of 1% to 4% reported in the literature (Hesse *et al.*, 2015 ▸; Palacio-Mancheno *et al.*, 2014 ▸; Yu *et al.*, 2020 ▸).

**Figure 9:**
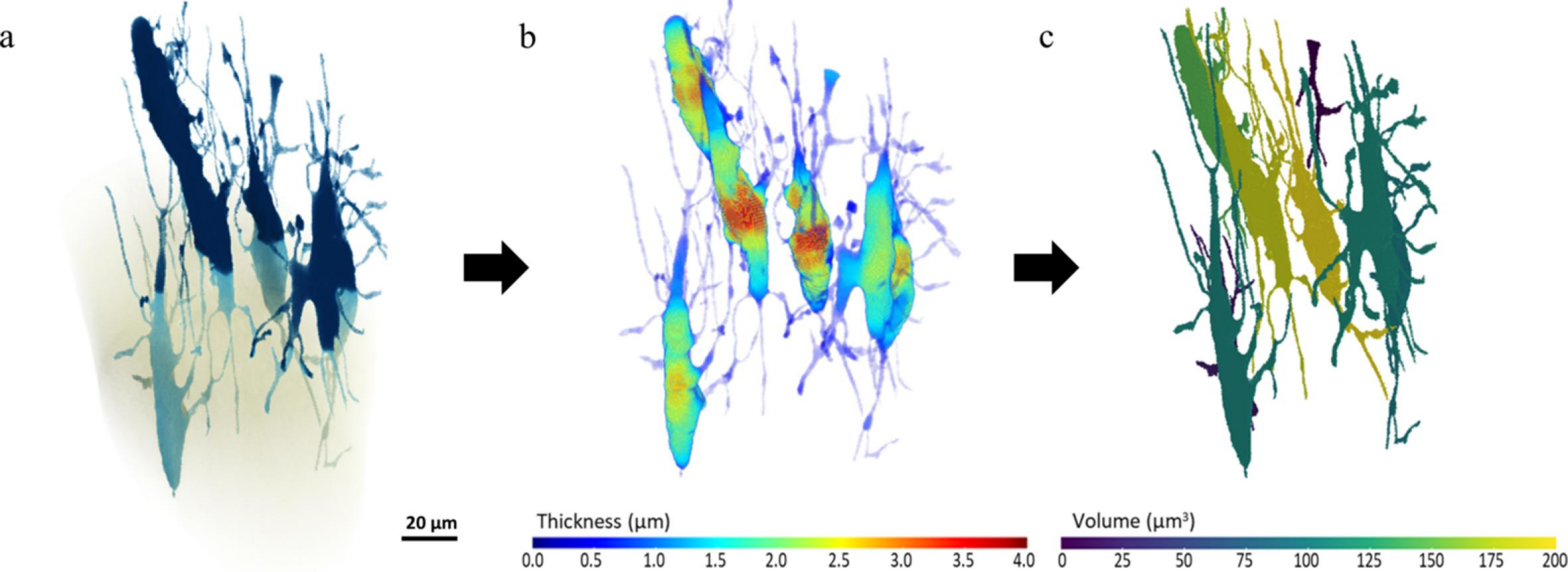
(*a*) 3D-rendering overview of the LCN within zebrafish spine bone imaged on the ANATOMIX beamline. The (*b*) thickness and (*c*) volume distribution can be calculated for each cell using available tools.

The LCN data can be further processed so that [Fig. 9 ▸(*a*)] each lacuna and its corresponding connected canaliculi can be measured for local thickness and volume. Fig. 9 ▸(*b*) shows that the 8 lacunae within the ANATOMIX dataset have a maximum thickness of 3.5 µm corresponding to previous reports of the diameters of lacunae voids (Carter *et al.*, 2013 ▸; McCreadie *et al.*, 2004 ▸; Yu *et al.*, 2020 ▸). Due to the non-linear phase-contrast enhancement Zernike effects, it is difficult to define the precise detection resolution for canaliculi. However, the effective pixel size sets a lower limit on what becomes visible with this method. The minimum canaliculi diameter detectable is limited by the spatial resolution of the scans, to ∼0.15 µm. Some individual lacunae with connected canaliculi have a volume above 150 µm^3^ [Fig. 9 ▸(*c*)]. Correctly classified pores in 3D makes it possible to further analyze the pore space distribution, pore paths and pore constrictions (Kollmannsberger *et al.*, 2017 ▸; van Tol *et al.*, 2020 ▸; Wittig *et al.*, 2019 ▸). Such data with quantitative estimates of the micro-nano void network, within relative sample volumes, are useful to characterize the fluid flow inside the LCN. This is useful for *e.g.* analyzing the transport of nutrients and waste products, in addition to being important for assessing poroelasticity and mechanotransduction due to deformation of the LCN across the bone (Cowin, 2002 ▸; Fritton & Weinbaum, 2009 ▸; van Tol *et al.*, 2020 ▸).

The ability of bone to adapt is believed to be regulated by the network of embedded osteocytes. In mammalian bone, the morphology of lacunae and their resident osteocytes are known to change with age and diseases. Analysis of Zernike-nanoCT data as described in the present work lays the groundwork for the geometric analysis, which may have implications for an improved understanding of the role of the LCN. We have proposed an end-to-end analysis procedure which encompasses image processing, segmentation and analysis of Zernike-nanoCT images of zebrafish bones. This will hopefully support ongoing efforts for the characterization of the interconnected porous system within bones (Varga *et al.*, 2015 ▸; Schneider *et al.*, 2007 ▸; Wittig *et al.*, 2019 ▸). The proposed DL approach is less time-consuming and more accurate than standard gray value or Otsu threshold methods and is likely to become a standard computer-aided segmentation approach well able to identify bone porosity in relatively large datasets of PCE tomographic data of bone with variable contrast.

## 5. Conclusions

The present work presents detailed steps needed to identify and quantify bone features by implementing a DL-based segmentation approach for 3D data with non-linear varying feature visibility. Zernike-nanoCT from both ANATOMIX and P05 beamlines provides high contrast and high spatial resolution, sufficient to reveal the micro-nano porosity of the LCN in zebrafish bone. However, there are limitations to the resolution and these define the smallest porosity detectable, suggesting that canaliculi smaller than 150 nm cannot be detected in this manner. The results here demonstrate that the DL models are a powerful tool for objective automatic segmentation of data with regionally varying contrast and therefore reproducible assessment of the interconnected porous systems embedded in X-ray Zernike phase-contrast imaged bone material. Nevertheless, differences in the signal-to-noise ratio of the data may affect the performance of different CNNs during training and may require larger amounts of data for robust classification.

## 6. Data availability

Trained models and raw data required to reproduce these results are available upon reasonable request by contacting Professor Paul Zaslansky (paul.zaslansky@charite.de).

## Supplementary Material

Supporting Figures 1-8 and Tables 1 and 2. DOI: 10.1107/S1600577523009852/mo5274sup1.pdf
