# Supplementary material for: Deep learning to overcome Zernike phase-contrast nanoCT artifacts for automated micro-nano porosity segmentation in bone
Source: J Synchrotron Radiat. 2024 Jan 1;31(Pt 1):136–49. doi: 10.1107/S1600577523009852 (PMC10833422; doi:10.1107/S1600577523009852)
Supplement: Supplementary file 1 [file s-31-00136-sup1.pdf]

JOURNAL OF SYNCHROTRON RADIATION

Volume 31 (2024)

Supporting information for article:

Deep learning to overcome Zernike phase-contrast nanoCT artifacts for automated micro-nano porosity segmentation in bone

Andreia Silveira, Imke Greving, Elena Longo, Mario Scheel, Timm Weitkamp, Claudia Fleck, Ron Shahar and Paul Zaslansky

Supplementary information

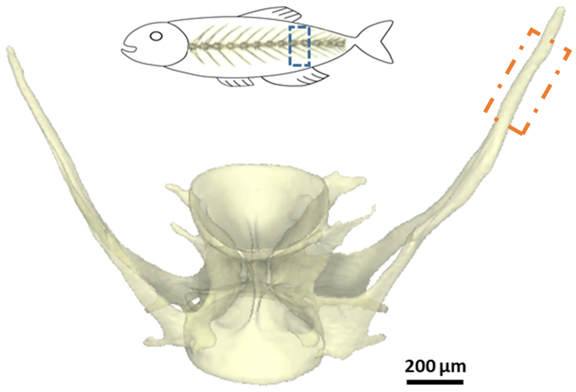

Fig. 1. 3D representation of a vertebra of the zebrafish skeleton within available micro-CT data of bone imaged on laboratory micro-computed tomography scanner (Skyscan 1172, Brucker-microCT Kontich, Belgium) as previously reported (Silveira *et al.*, 2022) with a pixel size of 5 µm. Part of Hemal spine (orange dashed rectangle) was imaged with Zernike nanoCT.

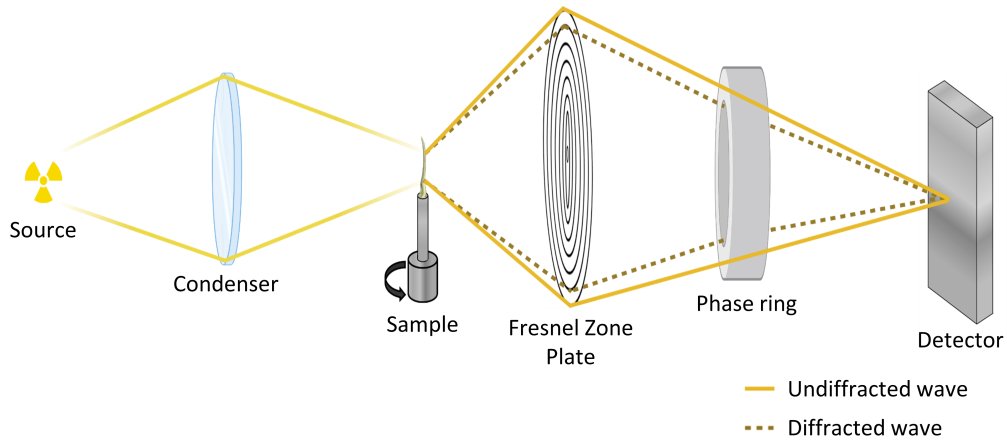

Fig. 2. Schematic representation of the experimental setup of the Zernike nano-CT experiment. Each spine of zebrafish vertebrae was mounted on the rotation stage. A parallel monochromatic X-ray beam is first converged onto the sample by the condenser lens, and then it divides into diffracted and undiffracted waves that pass through a Fresnel Zone Plate, which magnifies the sample image. The undiffracted wave is phase-shifted by the phase ring on the way to the detector. The image detector measures the sum of these two waves.

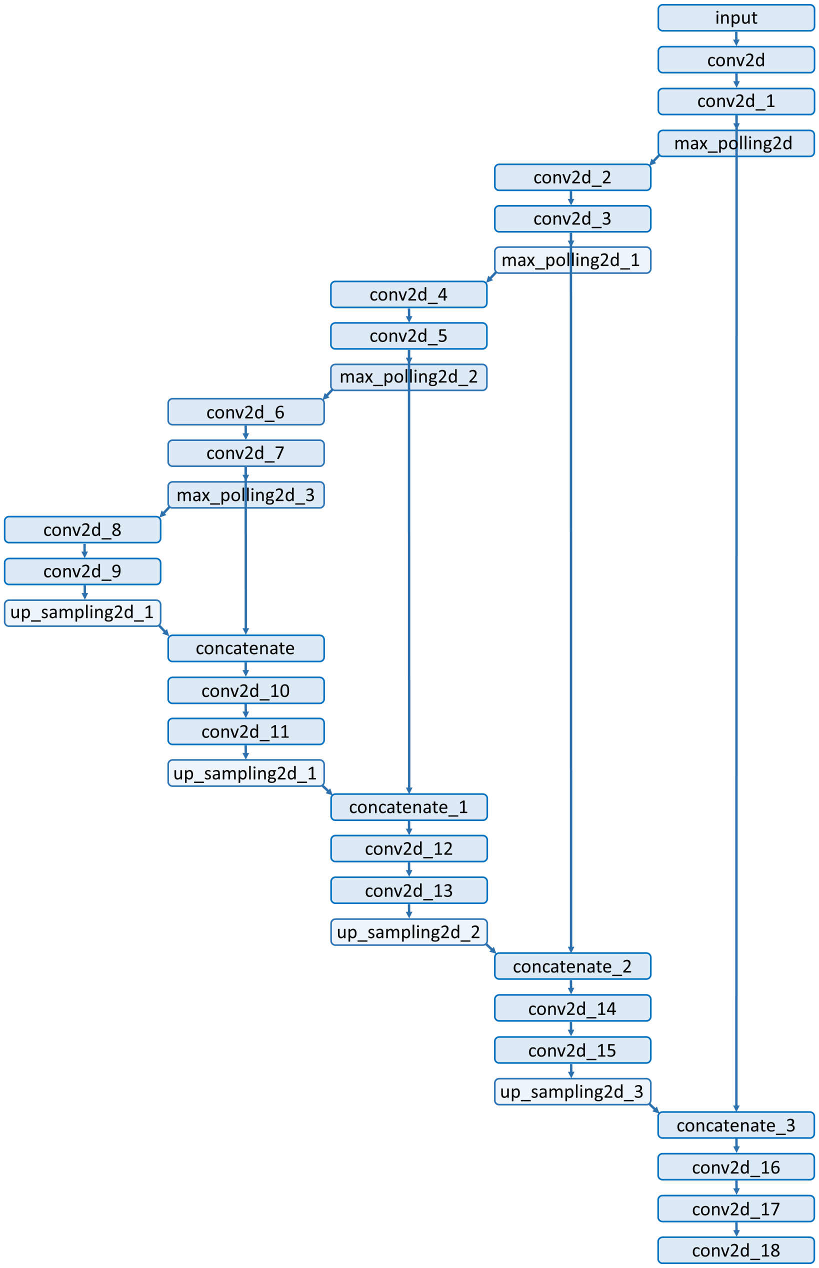

Fig. 3. Schematic diagram of the U-Net model architecture, extracted from the Dragonfly interface. It is based on (Ronneberger *et al.*, 2015) and is a widely implemented CNN. Briefly, the workflow contains encoder layers that capture contextual information in the data and reduce the spatial resolution of the input and decoder layers that will generate segmentation maps.

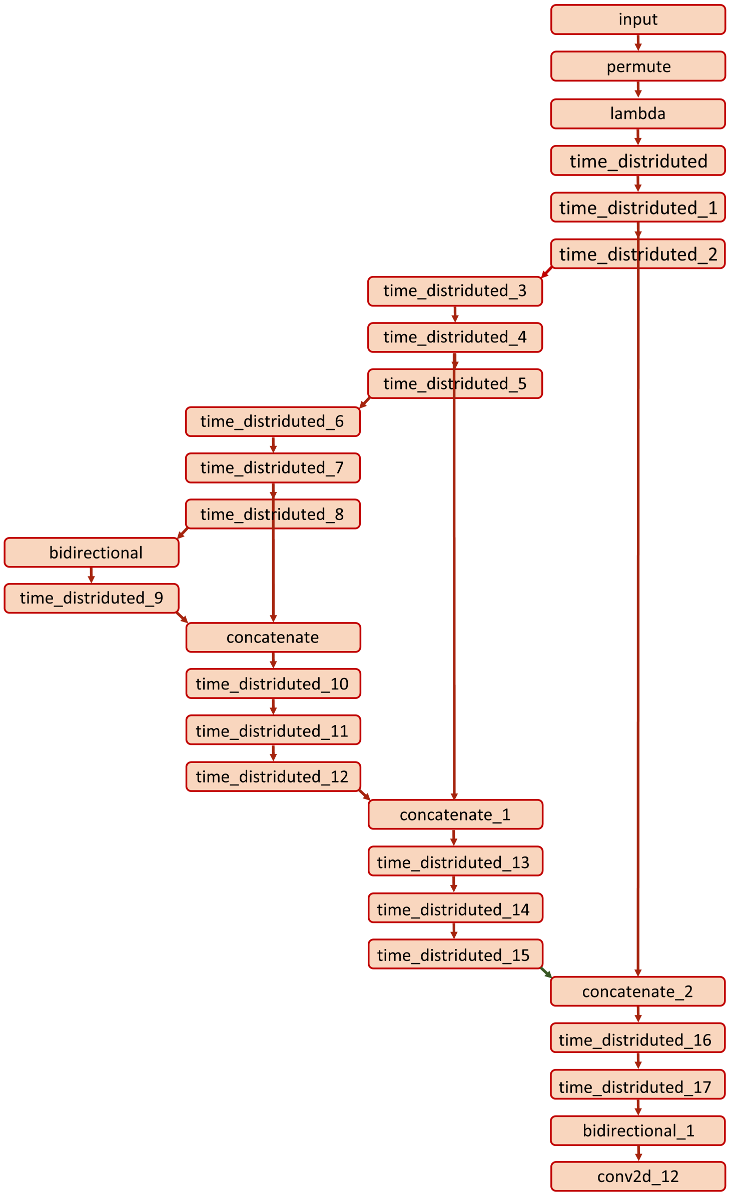

Fig. 4. Schematic diagram of the Sensor3D model architecture extracted from the Dragonfly interface. The architecture is based on details provided in (Novikov *et al.*, 2019) with modules that can be adapted in the Deragnofly interface. Briefly, this CNN combines time-distributed layers and bidirectional ConvLSTM (Long Short Term Memory) in an end-to-end U-Net-like hybrid architecture, yielding improved performance as compared to the U-Net architecture.

Table 1. *Model training for Anatomix dataset*

	Sensor3D				U-Net			
Batch size	32		64		32		64	
Trained size	Accuracy	Error Loss	Accuracy	Error Loss	Accuracy	Error Loss	Accuracy	Error Loss
20	0.933	0.067	0.908	0.092	0.938	0.062	0.933	0.067
50	0.943	0.057	0.945	0.055	0.957	0.043	0.952	0.048
70	0.968	0.032	0.947	0.053	0.960	0.040	0.950	0.050

Table 2. *Model validation for Anatomix dataset*

	Sensor3D				U-Net			
Batch size	32		64		32		64	
Trained size	Accuracy	Error Loss	Accuracy	Error Loss	Accuracy	Error Loss	Accuracy	Error Loss
20	0.896	0.104	0.816	0.184	0.898	0.101	0.812	0.188
50	0.906	0.094	0.934	0.066	0.917	0.0083	0.891	0.109
70	0.954	0.046	0.945	0.055	0.928	0.072	0.937	0.063

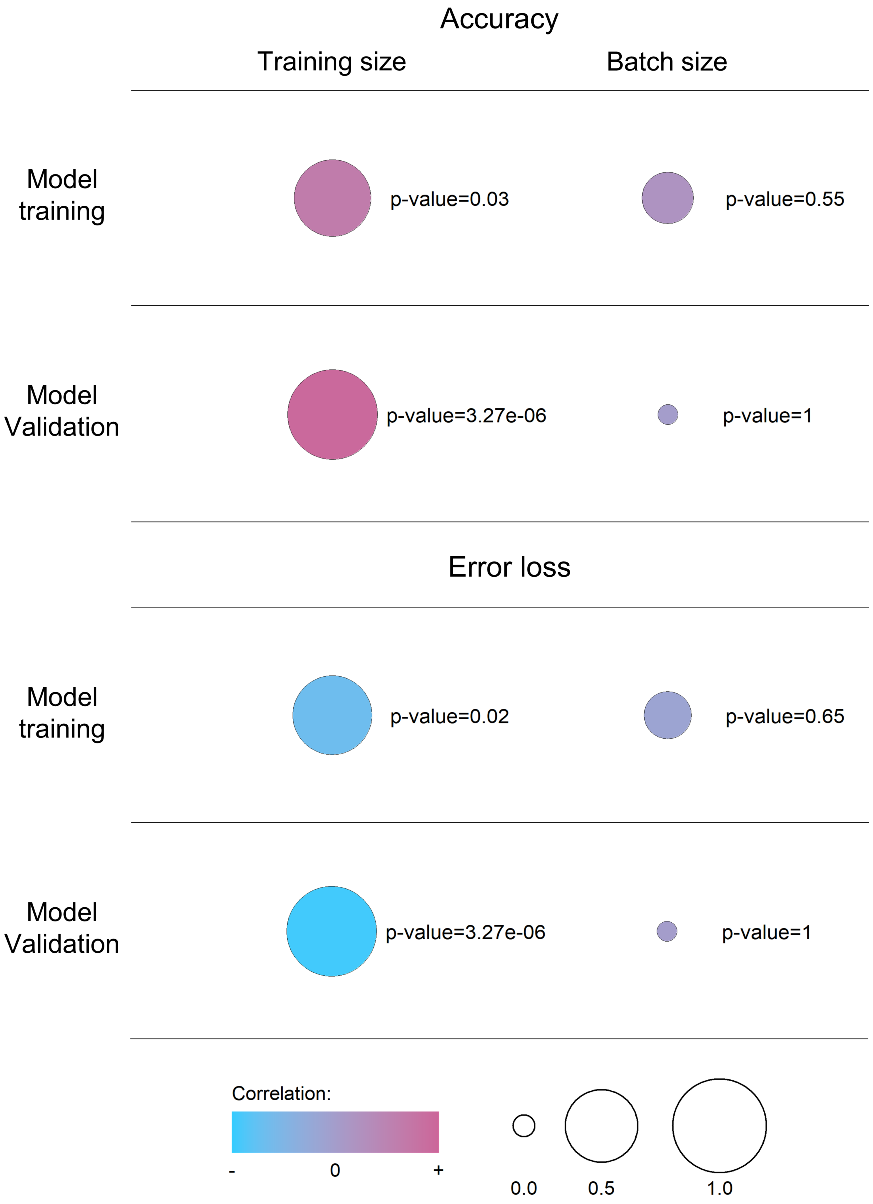

Fig. 5. Correlation between the training data size and batch size parameters and the accuracy (Dice coefficient) and error loss metrics (Categorical Cross-entropy loss function), used to evaluate the training and validation of the CNN models. The training data size presents a strong correlation, where the accuracy of the trained models increases with the increase of the training data size (positive correlation) and the error loss decreases with the increase of training data size (negative correlation). The batch size does not influence the accuracy and error loss of the trained models since it has a low correlation coefficient.

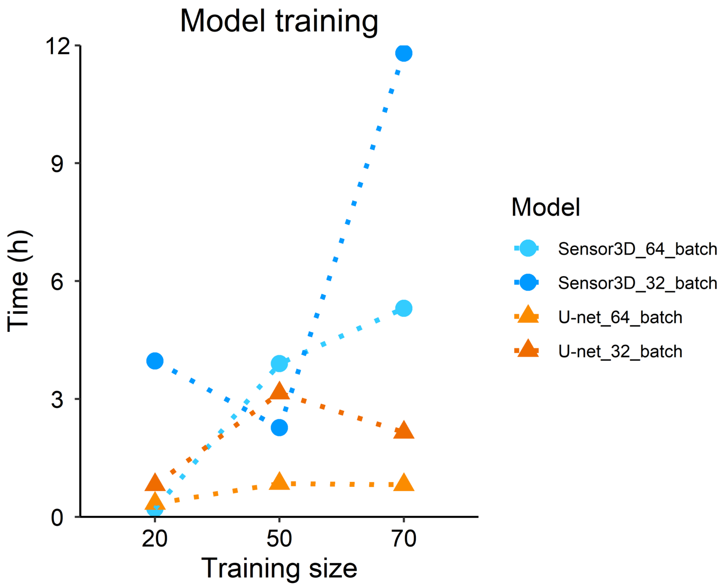

Fig. 6. Analysis of duration time to train the Sensor3D and U-Net models and the impact of the number of images used to train the models as well as the batch size used. Most of the models with a batch size of 64 took an average $\sim$ 150% less time to train than the models with a batch size of 32. But, in some cases, models trained with a batch size of 32 required fewer epochs and shorter training duration than models with a batch size of 64.

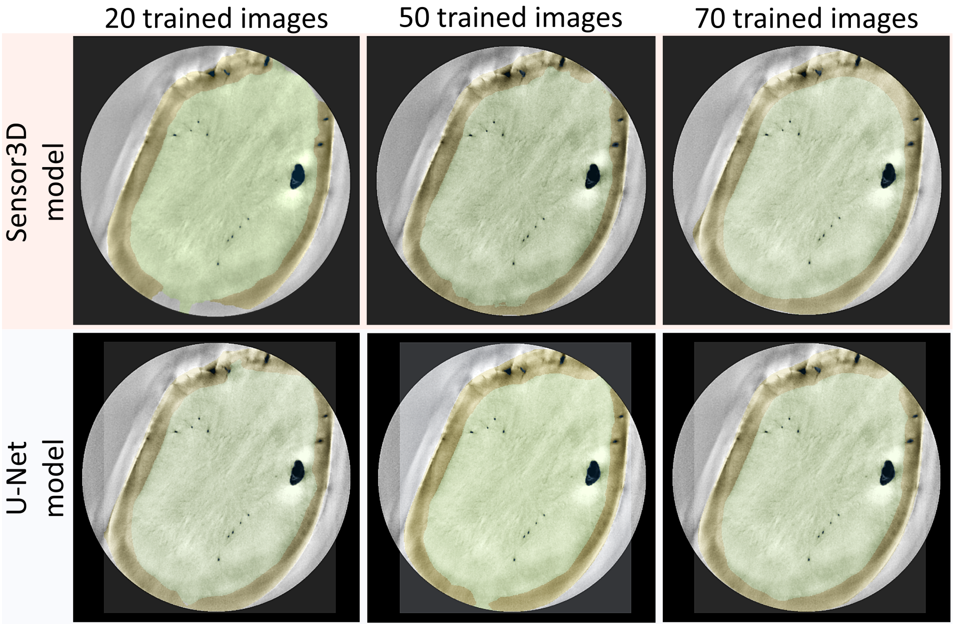

Fig. 7. Example slice of classification for shade-off (brown), bone (green) and the LCN (blue) classes comparing the result obtained for Sensor3D and U-Net models trained with 20, 50 and 70 ground truth images (training data size) at different stages. The models trained with 20 and 50 ground truth images have the worst outcome, mislabeling the bone class with background value or shade-off value. Both sensor3D and U-Net models trained with 70 ground truth images can correctly segment the voids, bone and shade-off regions.

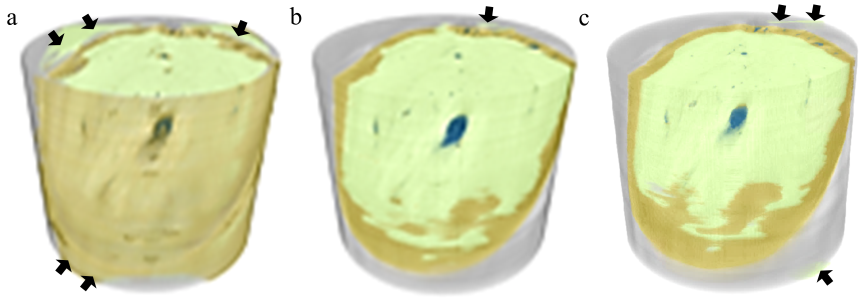

Fig. 8. Example renders comparing the resulted classification obtained with a) Otsu thresholding, b) Sensor3D model and c) U-net model for shade-off (brown), bone (green) and the LCN (blue) classes. The standard Otsu thresholding has the worst outcome and the trained Sensor3D model has the best outcome. The mislabeling pixels of bone class with background gray values are pointed out by the black arrows.

References

Novikov, A. A., Major, D., Wimmer, M., Lenis, D. & Buhler, K. (2019). *IEEE transactions on medical imaging*, **38**(5), 1207–1215.

Ronneberger, O., Fischer, P. & Brox, T. (2015). In *LNCS*, vol. 9351, pp. 234–241.

Silveira, A., Kardjilov, N., Markötter, H., Longo, E., Greving, I., Lasch, P., Shahar, R. & Zaslansky, P. (2022). *Materials & Design*, **224**, 111275.
URL: *https://www.sciencedirect.com/science/article/pii/S0264127522008978*
